# Prevalence and Impact of Chronic Liver Disease in Adult Patients Admitted With Cystic Fibrosis

**DOI:** 10.7759/cureus.101261

**Published:** 2026-01-10

**Authors:** Yifeng Yang, Weijia Li, Chengyue Jin

**Affiliations:** 1 Hospital Medicine, University of Texas Southwestern Medical Center, Dallas, USA; 2 Cardiology, Beth Israel Lahey Health, Inc., Cambridge, USA; 3 Helmsley Center for Electrophysiology, Icahn School of Medicine at Mount Sinai, New York City, USA

**Keywords:** chronic liver disease, cystic fibrosis (cf), large database analysis, patient outcomes research, quality improvement research

## Abstract

Background: Cystic fibrosis (CF) is a genetic, multisystem disorder characterized by progressive lung disease driven by mucus obstruction, chronic inflammation, and recurrent infections. Although chronic liver disease (CLD) is a recognized complication of CF, its prevalence and clinical impact among hospitalized adults with CF have not been well characterized using large-scale national databases.

Methods: We utilized the National Inpatient Sample (NIS) database to identify adults aged ≥18 years hospitalized between 2015 and 2019 with a diagnosis of CF. Patients were stratified based on the presence or absence of CLD. International Classification of Diseases (ICD) codes were used to identify comorbidities, including congestive heart failure, prior myocardial infarction, atrial fibrillation, obesity, chronic kidney disease, and end-stage renal disease. Multivariable regression analyses were performed to evaluate in-hospital mortality, length of stay, hospital costs, and in-hospital complications, including sepsis, pneumonia, acute kidney injury (AKI), and the need for intubation, tracheostomy, and gastrostomy.

Results: Among an estimated 90,675 hospitalized adults with CF, 11.68% had concomitant CLD. In multivariable analyses, patients with CLD had significantly higher in-hospital mortality compared with those without CLD (2.50% vs. 1.24%, P<0.001). The CLD group also experienced longer hospital stays (10.4 vs. 8.6 days, P<0.001) and higher hospitalization costs ($38,880 vs. $31,246, P=0.019).

The coexistence of CF and CLD was associated with a significantly increased risk of in-hospital complications, including sepsis (6.47% vs. 4.20%, P<0.001), AKI (17.13% vs. 11.79%, P<0.001), and malnutrition (42.63% vs. 31.49%, P=0.002). There were no significant differences between groups in the rates of pneumonia (21.00% vs. 20.57%, P=0.596), need for intubation (2.74% vs. 1.92%, P=0.139), tracheostomy (0.69% vs. 0.54%, P=0.072), or gastrostomy (0.89% vs. 0.66%, P=0.499).

Conclusions: CLD is common among adults hospitalized with CF and is independently associated with higher in-hospital mortality, longer length of stay, and increased hospital costs. Additionally, CLD confers a significantly higher risk of sepsis, AKI, and malnutrition. These findings underscore the importance of early recognition and proactive management of liver disease in hospitalized patients with CF.

## Introduction

Cystic fibrosis (CF) is the most common life-limiting autosomal recessive disorder among individuals of Caucasian descent, with an estimated global incidence of approximately one in 3,000 live births [1]. It is a multisystem disease that primarily affects the lungs, pancreas, sweat glands, and the Wolffian ducts in males [1]. In the United States, an estimated 33,288 individuals were living with CF, with adults accounting for 60.4% of the CF population, a substantial increase from 33.7% in 1993, reflecting improved survival and advances in care [2].

Liver disease is a well-recognized complication of CF; however, chronic liver disease (CLD) often presents insidiously or remains subclinical [1]. Most affected patients are asymptomatic and do not exhibit classic manifestations such as jaundice or pruritus. Furthermore, routine liver enzyme testing lacks adequate sensitivity and specificity for CF-related liver disease surveillance, making accurate estimation of CLD prevalence challenging [1, 3]. Consequently, reported prevalence rates vary substantially across studies, ranging from approximately 10% to 15% [2] to as high as 27% to 35% [4, 5].

CLD has been identified as an independent risk factor for mortality in patients with CF. A systematic review by Sasame et al. found that patients with CLD were at least three times the risk of death compared to those with no liver disease. They identified that pulmonary complications were the primary cause of death [6].

Despite the growing population of adults living with CF, large-scale database studies evaluating the prevalence and clinical outcomes of CLD in this population remain limited. A French cohort study by Boëlle et al. included approximately 4,000 patients with CF [7], while a nationwide UK study by Toledano et al. enrolled around 3,000 patients [8]. An Irish prospective longitudinal study included approximately 500 patients [9], and a large database analysis by Thavamani et al., although notable for its sample size, was restricted to pediatric patients and relied on a combination of International Classification of Diseases, Ninth Revision (ICD-9) and ICD-10 coding, which may limit generalizability to contemporary adult populations [10]. Collectively, these limitations underscore the need for comprehensive outcome studies that evaluate a broader, nationally representative hospitalized adult CF population.

In this study, we used data from the National Inpatient Sample (NIS) to evaluate the prevalence of CLD among adults hospitalized with CF and to examine its association with in-hospital clinical outcomes. The primary objective of this study was to evaluate in-hospital mortality, while the secondary objective was to assess in-hospital resource utilization, including hospital length of stay and total hospitalization costs, as well as in-hospital complications, including sepsis, pneumonia, acute kidney injury (AKI), and the need for mechanical ventilation, tracheostomy, and gastrostomy.

## Materials and methods

Patient database

We carried out a retrospective large-database analysis utilizing the National Inpatient Sample (NIS) [11]. The NIS is maintained by the Agency for Healthcare Research and Quality (AHRQ), and only anonymized data and materials were included in the database [11]. The NIS includes patient-level information with diagnosis and procedures, based on the ICD-10-Clinical Modification (ICD-10-CM) [12]. Encompassing hospitalizations from more than 4,000 non-federal hospitals across 45 states, the NIS included a 20% stratified sample of admissions to these hospitals and is designed to be an all-payer, nationally representative of the U.S. population [11]. As a de-identified, publicly available database conducted in compliance with the U.S. Health Insurance Portability and Accountability Act (HIPAA), the present study did not require local Institutional Review Board approval.

Study design

Using ICD-10 codes, we identified patients aged 18 years or older admitted with CF who were hospitalized between 2015 and 2019. We excluded patients younger than 18 years or older than 100 years, as well as those with missing values for key variables. We then stratified them into two groups based on the presence or absence of CLD [12]. We then compared the baseline demographic characteristics of patients with CLD and the control group, including individual-level characteristics like age, sex, and median household income, as well as hospital-level characteristics, including location, bed size, and teaching status [11]. We were also able to identify major comorbid conditions, including hypertension, diabetes, coronary artery disease, and obesity, and compared them between the two groups.

Our primary outcome was in-hospital mortality. Our secondary outcomes included measures of resource utilization, specifically hospital length of stay and total hospitalization costs, as well as in-hospital complications, including sepsis, pneumonia, AKI, and the need for mechanical ventilation (intubation), tracheostomy, and gastrostomy.

The ICD-10-CM codes used to identify the study population, comorbidities, and outcomes are detailed in Table 1 [12].

**Table 1:** International Classification of Diseases, Tenth Revision, Clinical Modification (ICD-10-CM) codes used to identify the study population, comorbidities, and in-hospital complications Diagnoses and procedures were identified using the ICD-10-CM [12]. Data were obtained from the National Inpatient Sample (NIS), Healthcare Cost and Utilization Project (HCUP), and Agency for Healthcare Research and Quality (AHRQ) [11].

Study population	ICD-10-CM codes
Cystic fibrosis	E84.x
Chronic liver disease	K70.x, K71.x, K72.x, K73.x, K74.x, K75.x, K76.x, and K77.x
Comorbidities	ICD-10-CM codes
Coronary artery disease	I20.x, I21.x, I22.x, I23.x, I24.x, I25.x
History of myocardial infarction	I25.2x
Hypertension	I10.x, I11.x, I12.x, I13.x, I15.x, I16.x
Diabetes mellitus	E08.x, E09.x, E10.x, E11.x, E13.x
Chronic kidney disease	N18.x
Atrial fibrillation/atrial flutter	I48.x
Peripheral artery disease	I73.89, I73.9x, I70.2x, I70.3x, I70.4x, I70.5x, I70.6x, I70.7x
Valvular heart disease	I34.x, I35.x, I36.x, I37.x, I38.x
Obesity	E66.x
Chronic obstructive pulmonary disease	J40.x, J41.x, J42.x, J43.x, J44.x, J47.x
History of stroke	I69.x
Congestive heart failure	I50.x
In-hospital complications	ICD-10-CM codes
Sepsis	A40.x, A41.x, R65.2x, T81.4x, T82.7x, A48.3x, B37.7x, A54.86
Acute kidney injury	N17.0x, N17.1x, N17.2x, N17.8x, N17.9x, N99.0x, N99.61, N99.71, N99.81, N99.820, N99.89
Intubation	0BH17EZ, 0BH18EZ (procedure codes)
Pneumonia	J12.x, J13.x, J14.x, J15.x, J16.x, J17.x, J18.x
Tracheostomy	Z93.0x, Z43.0x, J95.0x
Gastrostomy	Z93.1x, Z43.1x, K94.2x
Malnutrition	E40.x, E41.x, E42.x, E43.x, E44.x, E46.x

Statistical analysis

After 2012, the NIS uses a stratified sampling design based on hospital characteristics such as region, teaching status, bed size, and ownership. Within each stratum, all hospitals are included (100% sampling), and approximately 20% of discharges from each hospital are randomly selected for the dataset [11]. This approach ensures that the NIS remains nationally representative while keeping the dataset size manageable.

Given the complexity of its design, we applied appropriate discharge-level weights and survey-specific analytical methods, as used in our prior publications [13-15]. Specifically, strata were defined using a combined year-stratum variable (generated as egen STA = group(YEAR NIS_STRATUM)), and survey settings were specified using svyset [pweight = DISCWT], strata(STA) psu (HOSP_NIS). Survey-weighted analyses were conducted using the svy prefix with discharge weights (DISCWT) to generate nationally representative estimates [11].

Descriptive statistics were used to compare demographic characteristics, hospital characteristics, comorbidities, and outcomes between CF patients with and without CLD. Categorical variables were compared using the chi-square test (χ²), and continuous variables were compared using Student’s t-test. Multivariable regression analyses were performed to evaluate associations between CLD and study outcomes. Logistic regression was used for binary outcomes, and linear regression was used for continuous outcomes.

Covariates included in the multivariable models encompassed patient-level demographic factors (age, race, median annual income by patient ZIP code, and insurance type), hospital-level characteristics (hospital region and hospital bed size), and clinical comorbidities, including atrial fibrillation, hypertension, diabetes mellitus, congestive heart failure, history of stroke, valvular heart disease, coronary artery disease, peripheral artery disease, chronic obstructive pulmonary disease, history of myocardial infarction, obesity, and chronic kidney disease.

All analyses were conducted using Stata version 14 (StataCorp, College Station, TX). Statistical tests were two-sided, and a p-value < 0.05 was considered statistically significant.

## Results

Baseline characteristics

A total of 90,675 adult hospitalizations with a principal diagnosis of CF were identified. Baseline demographic, hospital, and clinical characteristics of patients with and without CLD are summarized in Table 2. Compared with the control group, patients with CLD were younger (mean age 30.1 ± 11.9 vs. 32.7 ± 13.7 years), more likely to be male and Hispanic, and more frequently insured by Medicare. Patients in the CLD group were also more likely to be admitted to large, urban teaching hospitals and to hospitals located in the Western region of the United States.

**Table 2:** Baseline characteristics of the control and chronic liver disease (CLD) groups *: According to Requirements for the HCUP Data Use Agreement, cells containing a value of 1 to 10 in both text and tables are replaced by *. Values represent weighted national estimates derived from the National Inpatient Sample (NIS), Healthcare Cost and Utilization Project (HCUP), and Agency for Healthcare Research and Quality (AHRQ) [11].

Variable	Control (%)	CLD group (%)	Chi-square test (χ²)	p-value
Number of patients	80084.2 (88.32%)	10590.8 (11.68%)		
Patient characteristics				
Women	44503.3 (55.58%)	4955.4 (46.77%)	97,400	<0.001
Race			38,700	0.0047
White	68142.3 (85.19%)	9149.1 (85.68%)		
Black	3833.7 (4.79%)	359.0 (3.36%)		
Hispanic	5727.9 (7.16%)	910.4 (8.52%)		
Asian or Pacific Islander	431.8 (0.54%)	31.2 (0.29%)		
Native American	353.7 (0.44%)	* (* %)		
Other	1503.4 (1.88%)	223.7 (2.09%)		
Mean age, years	32.7 (32.3-33.0)	30.1 (29.5-30.7)		<0.001
Median annual income in patients’ zip code, US$			11,000	0.2358
$1 - $38,999	18878.5 (23.58%)	2281.4 (21.51%)		
$39,000 - $47,999	21190.8 (26.47%)	2783.7 (26.24%)		
$48,000 - $62,999	21218.0 (26.49%)	2844.5 (26.82%)		
$63,000 or more	18787.9 (23.46%)	2697.6 (25.43%)		
Insurance type			31,800	0.0056
Medicaid	22505.5 (28.12%)	2793.7 (26.19%)		
Medicare	21109.1 (26.39%)	3269.7 (30.66%)		
Private	34746.7 (43.43%)	4435.8 (41.59%)		
Uninsured	1647.6 (2.06%)	165.8 (1.55%)		
Hospital characteristics				
Hospital region			73,500	0.0170
Northeast	13827.9 (17.27%)	1690.2 (15.95%)		
Midwest	20102.7 (25.1%)	2615.1 (24.68%)		
South	30330.8 (37.87%)	3560.0 (33.6%)		
West	15822.8 (19.76%)	2730.2 (25.7%)		
Hospital bed size			70,800	<0.001
Small	6920.3 (8.64%)	530.0 (5%)		
Medium	12930.3 (16.15%)	1499.8 (14.16%)		
Large	60226.3 (75.21%)	8565.2 (80.84%)		
Location of the hospital			34,500	<0.001
Rural hospital	2519.9 (3.15%)	145.0 (1.37%)		
Urban hospital	77563.4 (96.85%)	10445.8 (98.63%)		
Teaching status of the hospital			65,500	<0.001
Non-teaching hospital	7549.6 (9.43%)	560.0 (5.29%)		
Teaching hospital	72530.9 (90.57%)	10037.7 (94.71%)		
Comorbidities				
Atrial fibrillation	1529.7 (1.91%)	185.0 (1.75%)	453.40	0.6237
History of myocardial infarction	470.0 (0.59%)	25.0 (0.24%)	7054.45	0.0392
Obesity	2100.0 (2.62%)	260.0 (2.45%)	347.84	0.6587
Hypertension	13456.2 (16.8%)	1700.2 (16.05%)	1278.96	0.4479
Peripheral artery disease	245.0 (0.31%)	20.0 (0.19%)	1465.24	0.3530
Valvular heart disease	410.0 (0.51%)	55.0 (0.52%)	3.09	0.9650
Congestive heart failure	2509.9 (3.13%)	245.0 (2.31%)	7131.44	0.0415
Chronic obstructive lung disease	23466.7 (29.31%)	3164.6 (29.87%)	477.46	0.6668
Chronic kidney disease	7630.3 (9.53%)	820.0 (7.74%)	11,800	0.0204
History of stroke	360.0 (0.45%)	50.0 (0.47%)	34.57	0.8917
Coronary artery disease	1855.2 (2.32%)	180.0 (1.7%)	5404.92	0.0925
Diabetes mellitus	34728.5 (43.37%)	5964.6 (56.3%)	21,000	<0.001

With respect to comorbidities, patients with CLD were more likely to have diabetes mellitus but were less likely to have a history of myocardial infarction, congestive heart failure, or chronic kidney disease (Table 2). There were no significant differences between the CLD and control groups in median household income or in the prevalence of other comorbid conditions, including atrial fibrillation, obesity, and coronary artery disease.

Clinical outcomes

The unadjusted in-hospital mortality rate was higher among patients with CLD compared with those without CLD (2.5% vs. 1.24%). On multivariable regression analysis, CLD was independently associated with an increase in in-hospital mortality (adjusted odds ratio (OR) 2.22, 95% confidence interval (CI) 1.51-3.26; P < 0.001) (Table 3).

**Table 3:** Differences in in-hospital outcomes based on multivariable regression analysis between the chronic liver disease (CLD) group vs. control group All values are based on the CLD group vs. the control group. Multivariable regression analyses were performed using weighted data from the National Inpatient Sample (NIS), Healthcare Cost and Utilization Project (HCUP), and Agency for Healthcare Research and Quality (AHRQ) [11].

Outcome	Unadjusted incidence	Adjusted odds ratio (95% CI)	P value
Mortality	1.24% vs. 2.50%	2.22 (1.51-3.26)	<0.001
Sepsis	4.20% vs. 6.47%	1.78 (1.39-2.29)	<0.001
Acute Kidney Injury	11.79% vs. 17.13%	1.57 (1.31-1.87)	<0.001
Pneumonia	20.57% vs. 21.00%	1.04 (0.9-1.21)	0.596
Intubation	1.92% vs. 2.74%	1.31 (0.92-1.88)	0.139
Tracheostomy	0.54% vs. 0.07%	0.16 (0.02-1.18)	0.072
Gastrostomy	0.66% vs. 0.89%	1.3 (0.61-2.77)	0.499
Nonhome discharge	26.76% vs. 23.56%	0.94 (0.74-1.18)	0.574
Malnutrition	42.63% vs. 31.49%	1.37 (1.12-1.67)	0.002
Outcome	Unadjusted value (mean ± SD)	Regression coefficient (95% CI)	P value
Length of stay	8.6 ± 9.2 days vs. 10.4 ± 9.2 days	1.33 (0.79-1.88)	<0.001
Cost of hospital stay	$31,246 ± 70,043 vs. $38,880 ± 54,458	6398.76 (385.62-12411.9)	0.037

Major in-hospital complications were also more frequent in the CLD group (Table 3). After adjustment for potential confounders, CLD was independently associated with higher odds of sepsis (OR 1.78, 95% CI 1.39-2.29; P < 0.001), AKI (OR 1.57, 95% CI 1.31-1.87; P < 0.001), and malnutrition (OR 1.57, 95% CI 1.31-1.87; P < 0.001). In contrast, no significant differences were observed between the CLD and control groups with respect to pneumonia (OR 1.04, 95% CI 0.90-1.21; P = 0.596), need for intubation (OR 1.31, 95% CI 0.92-1.88; P = 0.139), tracheostomy (OR 0.16, 95% CI 0.02-1.18; P = 0.072), or gastrostomy (OR 1.30, 95% CI 0.61-2.77; P = 0.499).

Resource utilization

The unadjusted mean length of hospital stay was longer in the CLD group compared with the control group (10.4 ± 9.2 vs. 8.6 ± 9.2 days). On multivariable linear regression analysis, CLD was independently associated with an increased length of stay by approximately 1.33 days (regression coefficient 1.33; 95% CI 0.79-1.88; P < 0.001) (Table 3).

Similarly, patients in the CLD group incurred higher unadjusted mean hospitalization costs compared with controls ($38,880 ± 54,458 vs. $31,246 ± 70,043). After adjustment, CLD remained independently associated with higher hospitalization costs, with a regression coefficient of $6,398.76 (95% CI $385.62-$12,411.90; P = 0.037) (Table 3).

No significant difference in non-home discharge disposition was observed between patients with and without CLD (adjusted OR 1.04, 95% CI 0.93-1.15; P = 0.490).

## Discussion

In the present study, we used a national inpatient database to examine the prevalence and clinical impact of CLD among patients hospitalized with CF. We found that male sex and diabetes mellitus were associated with a higher risk of CLD, consistent with prior studies [7, 16]. On multivariable analysis, CLD was independently associated with higher in-hospital mortality and an increased risk of malnutrition, findings that align with previously published literature [7, 10, 17]. In addition, patients with CLD demonstrated increased healthcare resource utilization, including longer hospital stays and higher costs, similar to observations reported in pediatric CF populations [10].

Importantly, this study identifies several novel findings. We found that CLD was associated with significantly higher rates of sepsis and AKI, outcomes that have not been consistently reported in prior CF-related liver disease studies. Patients with comorbid CLD may be more immunocompromised due to cirrhosis-associated immune dysfunction, predisposing them to infections and sepsis. Furthermore, advanced liver disease can increase the risk of AKI through mechanisms such as hepatorenal syndrome, large-volume paracentesis, and aggressive diuresis. In contrast to a previous study, CLD was not associated with higher rates of tracheostomy or gastrostomy placement in our cohort [16]. This discrepancy may reflect differences in study populations, as prior studies may have included patients with more advanced or severe liver disease.

These findings highlight the importance of early recognition and vigilant monitoring for liver disease in patients with CF. Given its often subtle and asymptomatic presentation, routine surveillance strategies, including liver function tests, hepatic ultrasound, and noninvasive fibrosis assessment using the FIB-4 index and transient elastography, should be considered during hospitalization [18]. Particular attention should be given to high-risk populations, including male patients and those with a history of meconium ileus, pancreatic insufficiency, chronic colonization with *Burkholderia cepacia*, and frequent intravenous antibiotic exposure, all of which have been independently associated with CF-related liver disease [17]. While no specific CFTR mutations have been consistently linked to liver disease severity, environmental and genetic modifiers, such as the SERPINA1 Z allele, may play an important role in disease development [16].

This study has several limitations. First, the NIS relies on ICD codes, limiting access to detailed clinical data such as laboratory values, imaging findings, medication use, and physical examination results. Identification of CLD was based solely on ICD coding, which may have resulted in misclassification or underdiagnosis. Additionally, we were unable to assess the severity of CLD, which may have influenced comparisons of clinical outcomes, healthcare utilization, hospital costs, and length of stay. The lack of granular clinical data also precluded evaluation of disease progression or management changes related to CLD. Due to the inherent characteristics of the NIS database, the findings of this study are limited to hospitalized patients with CF. Given the retrospective and observational nature of the study, only associations could be assessed, and causality cannot be inferred. Finally, despite multivariable adjustment, residual confounding inherent to administrative database studies may have influenced the observed associations.

## Conclusions

CLD is common among adults hospitalized with CF and is independently associated with higher in-hospital mortality, longer length of stay, and increased hospital costs. Additionally, CLD confers a significantly higher risk of sepsis, AKI, and malnutrition in hospitalized patients with cystic fibrosis.

Although ursodeoxycholic acid may not alter the incidence of advanced liver disease, it may still be considered in patients diagnosed with CLD. These findings underscore the importance of early recognition and proactive management of liver disease in hospitalized patients with CF.
